# Assessing seropositivity of MMR antibodies in individuals aged 2–22: evaluating routine vaccination effectiveness after the 2003 mass campaign-a study from Iran’s National Measles Laboratory

**DOI:** 10.1186/s12879-024-09593-6

**Published:** 2024-07-12

**Authors:** Fateme Ghafoori, Talat Mokhtari-Azad, Abbas Rahimi Foroushani, Mohammad Farahmand, Azade shadab, Vahid Salimi

**Affiliations:** 1 Department of Virology, School of Public Health, Tehran University of Medical Sciences, Poursina Ave, Qods St, Enqelab Sq, Tehran, Iran; 2 National Measles and Rubella Lab of Iran, Tehran, Iran; 3 Department of Epidemiology and Biostatistics, School of Public Health, Tehran University of Medical Sciences, Tehran, Iran; 4 Pediatric Infectious Disease Research Center, Tehran University of Medical Sciences, Tehran, Iran

**Keywords:** Measles, Mumps, Rubella, Antibody status, Seroprevalence, Vaccine

## Abstract

**Background and purpose:**

The seroprevalence of antibodies against measles, mumps, and rubella (MMR) was evaluated 17 years following a mass vaccination campaign in individuals aged 2 to 22 years who had received routine immunization but were not eligible for an extended immunization program.

**Methods:**

Samples were acquired from Iran’s National Measles Laboratory (NML), with individuals showing positive IgM results excluded. Out of the samples collected in 2020, a random selection of 290 serum samples was chosen, representing individuals between the ages of 2 and 22 years from diverse regions in the country. These samples were subjected to analysis using an enzyme-linked immunosorbent assay (ELISA) to quantify specific IgG antibodies against MMR.

**Results:**

The seroprevalence rates of antibodies for measles, mumps, and rubella were determined to be 76.2%, 89.3%, and 76.9%, respectively. Younger age groups exhibited higher seropositivity rates for measles and mumps, whereas the 7- to 11-year-old group demonstrated the highest seropositivity rate for rubella. A reduction in antibody status was observed from younger to older age groups, particularly those aged 17–22.

**Conclusion:**

The study unveiled suboptimal antibody levels for measles and rubella, highlighting the necessity for further investigation and potential adjustments to future vaccination strategies. Moreover, the decline in antibody status post-vaccination can accumulate in seronegative individuals over time, elevating the risk of outbreaks.

## Introduction

For the past two decades, Iran has been making efforts to eliminate highly contagious diseases such as measles, mumps, and rubella (MMR), which can have long-term complications and even lead to death. Previously, children in Iran received routine vaccinations for measles at ages 9 and 15 months [1, 2]. In 2003, an extended immunization program was introduced, including MR vaccinations for individuals aged 5 to 25. Following the implementation of this program, there was a significant increase in herd immunity, resulting in a noticeable decrease in the number of measles outbreaks across the country [2]. However, since 2004, the vaccination schedule has been altered to administer shots of the measles-rubella-mumps (MMR) vaccine at 12 months and between 4 and 6 years of age. The vaccination schedule was modified in 2008 to administer shots at 12 and 18 months of age [1]. Fortunately, the Islamic Republic of Iran received a certificate of measles elimination in the WHO Eastern Mediterranean Region (EMRO) based on the Regional Verification Committee (RVC) on May 28, 2019 [3].

Although the average immunization coverage of entire population in Iran is estimated to be approximately 95% [1–3], it should be noted that this alone is not enough to determine community immunity levels. To obtain a comprehensive overview of the effectiveness of the immunization program, it is crucial to integrate immunization coverage data with antibody prevalence rates. Doing so can help identify gaps in population immunity [4, 5]. This study aims to evaluate the prevalence rates of antibodies against measles, mumps, and rubella among people aged 2 to 22 who were vaccinated through the routine immunization program but did not participate in the 2003 Expanded Program on Immunization (EPI). This research could provide insight into the effectiveness of Iran’s national immunization schedule 17 years after the mass vaccination campaign.

## Methods

### Sampling scheme

The research concentrated on individuals aged 2 to 22 who were not part of Iran’s 2003 mass vaccination campaign. Samples were procured from Iran’s National Measles Laboratory (NML), and those exhibiting positive IgM results were excluded. Out of the pool of samples collected in 2020, 290 serum samples were randomly chosen to represent individuals aged 2–22 nationwide. These participants were stratified into four age subgroups: 2–6, 7–11, 12–16, and 17–22. All pertinent information regarding the analyzed samples is derived from the database of the National Measles Center of Iran.

#### Questionnaire

The questionnaire employed in the study included inquiries about dates of birth, previous occurrences of measles, mumps, rubella, and the number of vaccination doses administered. The vaccination history was corroborated using the participants’ immunization booklets. In certain instances, the history of MMR vaccination was based on the participants’ statements.

### Laboratory procedure

The blood samples were taken to a local laboratory at the district level, where the sera were separated and stored at -20 °C until they were delivered to the National Reference Measles Laboratory at Tehran University of Medical Sciences in Tehran, Iran. All serum samples used for the test had the appropriate volume and were in good condition, with no hemolysis or lipemic specimens included.

To measure the presence of IgG antibodies against MMR, an indirect ELISA was performed following the instructions provided by the manufacturer (EUROIMMUN Anti-Rubella Virus ELISA (IgG), Anti-Measles Virus ELISA (IgG), and Anti-Mumps Virus ELISA (IgG), Germany). The levels of IgG antibodies were classified into three groups according to the kit instructions: measles-seropositive (≥ 275 IU/mL), seronegative (< 200 IU/mL), and borderline (< 275 and ≥ 200 IU/mL); for mumps - seropositive (≥ 22 IU/mL), seronegative (< 16 IU/mL), and borderline (< 22 and ≥ 16 IU/mL); and for rubella - seropositive (≥ 11 IU/mL), seronegative (< 8 IU/mL), and borderline (< 11 and ≥ 8 IU/mL).

According to the manufacturer’s report, the kits had a specificity and sensitivity of 100% for measles and mumps and 100% and 99.6% for rubella, respectively. Borderline results were retested using the ELISA method and the Vircell Microbiology ELISA measles IgG/IgM, rubella, and mumps IgG kits (Vircell, S. L. Parque Technologico de la salud. Avecina 8, 18,016 Granada, Spain).

Following the protocol of the Vircell kit, seronegative and borderline results were considered seronegative cases.

#### Statistical analysis

The frequencies and percentages were used to express the categorical variables, and the chi-square test was used to test the association of antibody status with age and vaccination dose. A p-value below 0.05 was deemed significant. All statistical analyses were conducted using IBM SPSS version 21 in Armonk, NY, USA, and Graph Pad Prism 8 software based in La Jolla, CA, USA. Additionally, graphs were created using Microsoft Office Excel 2013.

## Results

The study involved 290 samples, and the frequency and relative frequency by age and gender are presented in Table 1. Of the entire cohort, 123 individuals (42.4%) were male, while 167 individuals (57.6%) were female. Among the various age groups, the largest contingent, comprising 120 individuals (41.4%), belonged to the 7–11 age group. The 2–6 age group, consisting of 98 individuals, constituted the second-largest group, accounting for 33.8%.

The geometric mean levels for females were as follows: antimeasles − 554.9 mIU/mL, antimmumps − 71.6 mIU/mL, and antirubella − 58.82 mIU/mL. The geometric mean levels among males were slightly lower: antimeasles − 468.4 mIU/mL, antimmumps − 67.08 mIU/mL, and antirubella − 47.51 mIU/mL. Regarding age groups, participants aged 2–6 years exhibited the highest geometric mean levels for all three antibodies: antimeasles − 739.5 mIU/mL, antimmumps − 93.91 mIU/mL, and antirubella − 43.61 mIU/mL. The geometric mean levels gradually decreased with increasing age groups, with participants aged 17–22 years having the lowest levels: antimeasles − 400.8 mIU/mL, antimmumps − 38.94 mIU/mL, and antirubella − 31.77 mIU/mL.

Additionally, Table 2 provides information on the antibody status against measles, mumps, and rubella for different age groups. The study revealed variations in antibody status to measles, mumps, and rubella across different age groups. For measles, the highest antibody level was observed in the age group of 2–6 years (81.6%), while the lowest was in the age group of 17–22 years (58.8%). Overall, 76.2% of individuals tested seropositive for measles. Furthermore, for mumps, the highest antibody level was again in the age group of 2–6 years (93.9%), with the lowest observed in the age group of 17–22 years (76.5%). Overall, 89.3% of individuals had positive antibody status for mumps. Moreover, for rubella, the highest antibody status was in the age group of 7–11 years (85.8%), and the lowest was in the age group of 17–22 years (64.7%). Overall, 76.9% of individuals were seropositive for rubella.

The study results suggest that younger individuals generally exhibit higher antibodies against measles, mumps, and rubella levels than their older counterparts.

**Fig. 1:**
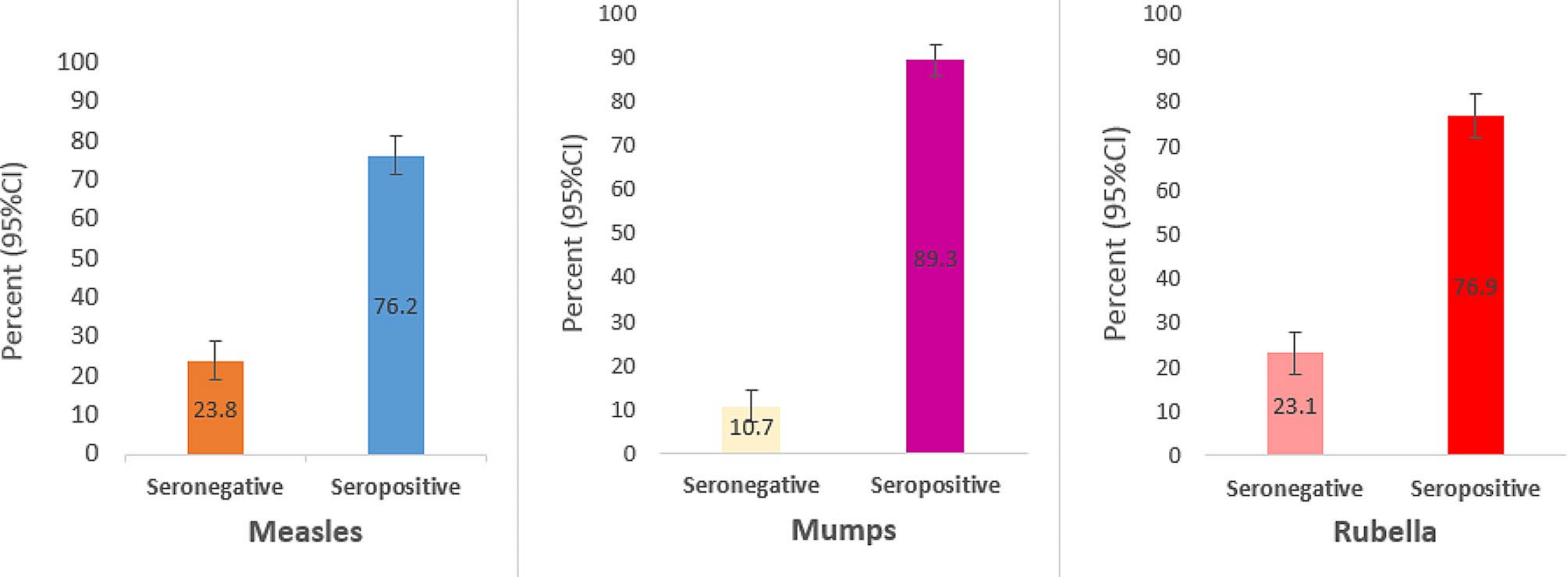
Antibody status to measles, mumps and rubella among people aged 2 to 22 who were not attended in mass campaign 2003 in 2020 in Iran

Table 3 presents the seroprevalence of anti-measles, anti-mumps, and anti-rubella in subjects categorized by the number of MMR vaccine doses they received. Information regarding the vaccination history of eight individuals was unavailable, and consequently, they were excluded from the calculations. Among those who had not received any vaccine for measles, mumps, and rubella, the lowest antibody levels based on the dose number were 66.7%, 71.4%, and 76.2%, respectively. On the other hand, the highest levels of antibodies to measles and rubella were observed in individuals who had received a single dose of the vaccine (79.4% for both), while the highest level of antibody to mumps (91.8%) was seen in individuals who had received two doses of the vaccine. The seroprevalence rates for measles, mumps, and rubella were 76.2%, 89.3%, and 76.9%, respectively (Fig. 1).

**Table 1:** Descriptive based on population characteristics among 2- to 22-year-olds who did not attend mass campaign 2003 in 2020 in Iran

variable		Frequency *N* (%)	Geometric Mean Antimeasles (mIU/mL)	Geometric Mean Antimmumps (mIU/mL)	Geometric Mean Antirubella (mIU/mL)
Gender	Female	167 (57.6)	554.9 (SD: 3.18)	71.6 (SD: 2.82)	58.82 (SD: 5.39)
	Male	123 (42.4)	468.4 (SD: 3.18)	67.08 (SD: 2.56)	47.51 (SD: 7.63)
Age group (Years)	2–6	98 (33.8)	739.5 (SD: 3.27)	93.91 (SD: 2.53)	43.61 (SD: 8.81)
	7–11	120 (41.4)	416.6 (SD: 2.94)	62.88 (SD: 2.4)	71.88 (SD: 5.16)
	12–16	55 (19)	416.8 (SD: 2.96)	58.26 (SD: 2.86)	39.97 (SD: 5.77)
	17–22	17 (5.9)	400.8 (SD: 3.89)	38.94 (SD: 3.62)	31.77 (SD: 8.90)
Total		290 (100)			

**Table 2:** Antibody status against measles, mumps, and rubella in different age groups in 2-22-year-olds who were not attended in mass campaign 2003 in Iran in 2020

Age group by year	Antibody status of Measles		Total	P value*	Antibody status of Mumps		Total	P value*	Antibody status of Rubella		Total	P value*
	Seronegative N (%)	Seropositive N (%)			Seronegative N (%)	Seropositive N (%)			Seronegative N (%)	Seropositive N (%)		
2–6	18 (18.4)	80 (81.6)	98 (100)	0.180	6 (6.1)	92 (93.9)	98 (100)	0.072	34 (34.7)	64 (65.3)	98 (100)	0.002
7–11	28 (23.3)	92 (76.7)	120 (100)		12 (10)	108 (90)	120 (100)		17 (14.2)	103 (85.8)	120 (100)	
12–16	16 (29.1)	39(70.9)	55 (100)		9 (16.4)	46 (83.6)	55 (100)		10 (18.2)	45 (81.8)	55 (100)	
17–22	7 (41.2)	10 (58.8)	17 (100)		4 (23.5)	13 (76.5)	17 (100)		6 (35.3)	11 (64.7)	17 (100)	
Total	69 (23.8)	221 (76.2)	290 (100)		31 (10.7)	259 (89.3)	290 (100)		67 (23.1)	223 (76.9)	290 (100)	

*chi-square test

**Table 3:** Measles, Mumps, and Rubella antibody status by number of MMR vaccine doses among 2–22 years old who were not attended in mass campaign 2003 in Iran in 2020

Vaccine Doses	Antibody status to Measles		Total	P value*	Antibody status to Mumps		Total	P value*	Antibody status to Rubella		Total	P value*
	Seronegative N (%)	Seropositive N (%)			Seronegative N (%)	Seropositive N (%)			Seronegative N (%)	Seropositive N (%)		
0	9 (42.9)	12 (57.1)	21 (100)	0.222	6 (28.6)	15 (71.4)	21 (100)	0.016	5 (23.8)	16 (76.2)	21 (100)	0.819
1	16 (23.5)	52 (76.5)	68 (100)		7 (10.3)	61 (89.7)	68 (100)		14 (20.6)	54 (79.4)	68 (100)	
2	53 (27.5)	140 (72.5)	193 (100)		16 (8.4)	175 (91.6)	191 (100)		47 (24.4)	146 (75.6)	193 (100)	
Total	78 (27.7)	204 (72.3)	282 (100)		29 (10.4)	251 (89.6)	280 (100)		66 (23.4)	216 (76.6)	282 (100)	

*chi-square test

## Discussion

Limited information exists regarding the efficacy of routine vaccination programs following the mass campaign 2003, which administered the measles and rubella (MR) vaccine to individuals aged 5 to 25 years in Iran. Thus, the primary objective of this survey was to assess the seropositive rate of MMR within the age range of 2–22 years in 2020. Table 2 illustrates a decline in antibody status against measles and mumps with age, particularly in the 17- to 22-year-old group. Similarly, the 2- to 6-year-old and 7- to 22-year-old groups displayed lower antibody levels for rubella, as indicated in Table 2. The diminishing antibody levels may also reduce antibody levels among older age groups. Previous surveys conducted in Portugal and Korea have highlighted the significance of waning antibody status in the declining trend of antibody levels [4, 5]. Among these age groups, 17-22-year-olds, especially women of childbearing age, are particularly concerned about rubella. A prior survey in Iran demonstrated a declining trend in seroimmunity for rubella and measles, which could be attributed to waning immunity [6]. Furthermore, the COVID-19 pandemic has reduced participation in routine vaccination programs, potentially leading to reduced immunity against rubella among those aged 2–6 years [7].

Table 3 reveals that the levels of antibodies against measles and rubella did not significantly differ between different doses of the MMR vaccine. However, individuals who had not received the MMR vaccination had the lowest antibodies to measles and rubella, while those who had received a single dose exhibited the highest antibody levels. Table 3 displays seroimmunity rates for anti-mumps based on the dose of the MMR vaccine administered. Individuals who had received two doses of the MMR vaccine exhibited the highest antibody levels for mumps, whereas those who had not received any dose of the vaccine had the lowest levels. Moreover, this relationship was statistically significant. It is essential to note that the study’s findings revealed lower antibody levels against measles and rubella in individuals who had received two doses of the vaccine than those who had received just one, possibly due to the small sample size or even primary vaccination failures.

In contrast to the results of the current study, a survey conducted in Iran demonstrated that the number of measles cases was lower among individuals who had received two doses of the MMR vaccine than among those who had received only one dose [8]. The primary limitation to the generalization of these results is that the study’s samples from the National Measles Laboratory may not fully represent the entire population, and therefore, the overall seroprevalence status of the community may not be accurately reflected. More extensive community-based studies are needed to more effectively assess the seroprevalence status within the target age range.

Previous studies also reported similarly low seroimmunity rates for measles and mumps, with some outbreaks occurring even among individuals who had received two prior doses of the MMR vaccine [3, 9–12]. No vaccine is entirely practical, and the MMR vaccine is no exception. Approximately 2–10% of vaccinated individuals may remain seronegative, but nearly 95% will respond to the vaccine if administered after one year of age [10, 13].

Seroepidemiological studies in Iran have revealed a disparity between immunization coverage and protective antibody levels for measles in the population. This discrepancy can be attributed to various factors, including the age at vaccination initiation, the interval since the last dose, vaccine strain, and the maintenance of the cold chain in healthcare facilities [14]. The risk of contracting measles was significantly associated with the age at which the first dose of the vaccine was administered. Lower efficacy and immunogenicity were observed when the initial dose was given before 12 months of age [6, 8, 15]. The effectiveness of the MMR vaccine dramatically depends on the timing of the first dose, as it plays a crucial role in establishing long-lasting immunity [16]. In Iran, from 2008 until now, the first dose has typically been administered at one year, followed by the second dose at 18 months as part of the national immunization program. For subjects aged 2 to 12, two doses of the MMR vaccine were administered at 12 and 18 months. However, those between 13 and 16 years old received their doses at 12 months and 6 years of age. Subjects aged 17 to 22 received two doses at 9 and 15 months. A survey conducted on individuals who received two MMR vaccine doses revealed that those who received their first dose before 13 months of age had a heightened risk of measles compared to those who received it after 15 months. Another study found that the rate of primary vaccination failure for measles, mumps, and rubella after the first MMR dose in Iran was higher than that in other regions across the globe [17].

According to research, the minimum levels of antibody protection needed for measles, mumps, and rubella are approximately 93–95%, 86–93%, and 83–94%, respectively [14, 18, 19]. In this study, the antibody status against mumps was found to be satisfactory, but the levels of antibody status against measles and rubella could be below the required threshold for sustaining elimination. However, it is essential to consider several factors before drawing any conclusions. First, ELISA may not accurately measure antibody status, as it has been found to have a false-negative rate of 10% or more when compared to the more reliable plaque reduction neutralization tests [20–22]. Second, it is essential to mention that Iran has a population of 87 million, and approximately 65% of individuals fall outside the age group included in the study.

Therefore, a thorough investigation is required to evaluate the actual level of antibody for these viruses within society. Third, the role of other immune mechanisms, such as cell-mediated immunity, should be considered [23–25]. Cellular immunity is significant because individuals with low levels of specific antibodies can still have sufficient responses when exposed to the virus [4]. Therefore, in communities with high vaccination rates, the herd immunity to MMR may be higher than indicated solely by serological test results.

Additionally, it is essential to mention that the level of community antibody status could potentially be higher than that observed in the study group. This can be attributed to individuals in the community infected with the wild virus, resulting in a reinforced immune response. The decline in seroprevalence observed results from decreasing vaccine-induced antibodies, which are not boosted naturally. Therefore, in the future, it may be necessary to administer supplementary vaccinations as part of routine vaccination programs to counteract the decreasing antibody levels.

## Conclusions

The research indicates that, despite the high vaccination coverage of the MMR vaccine in Iran, the population under study may exhibit insufficient antibodies against measles and rubella to sustain the elimination of these diseases. However, the antibody status for mumps was found to be satisfactory. Furthermore, an intriguing observation pertains to women aged 17 to 22, within the childbearing age bracket, demonstrating lower antibody levels against rubella. This highlights the need for the identification and vaccination of individuals at risk prior to pregnancy. The waning of antibody levels post-vaccination can accumulate seronegative individuals over time, elevating the potential for outbreaks.

Consequently, administering additional vaccine doses to older age groups is recommended to bolster herd immunity levels and deter the transmission of these viruses. Additionally, it is crucial to consider other immune mechanisms, such as cell-mediated immunity, when assessing the population’s overall immunity. Future consideration of supplementary vaccinations may be necessary due to the declining antibody levels attributed to routine vaccination programs.

## Data Availability

The data that support the findings of this study are available from the corresponding author upon reasonable request.
